# Effect of Obesity on Acute Ozone-Induced Changes in Airway Function, Reactivity, and Inflammation in Adult Females

**DOI:** 10.1371/journal.pone.0160030

**Published:** 2016-08-11

**Authors:** William D. Bennett, Sally Ivins, Neil E. Alexis, Jihong Wu, Philip A. Bromberg, Sukhdev S. Brar, Gregory Travlos, Stephanie J. London

**Affiliations:** 1 Center for Environmental Medicine, Asthma and Lung Biology, University of North Carolina at Chapel Hill, Chapel Hill, North Carolina, United States of America; 2 Division of Intramural Research, National Institute of Environmental Health Sciences, National Institutes of Health, Department of Health and Human Services, Research Triangle Park, North Carolina, United States of America; Leids Universitair Medisch Centrum, NETHERLANDS

## Abstract

We previously observed greater ozone-induced lung function decrements in obese than non-obese women. Animal models suggest that obesity enhances ozone-induced airway reactivity and inflammation. In a controlled exposure study, we compared the acute effect of randomized 0.4ppm ozone and air exposures (2 h with intermittent light exercise) in obese (N = 20) (30<BMI<40Kg/m2) vs. non-obese (N = 20) (BMI<25Kg/m2) non-smoking 18–35 year old women. We measured spirometry and bronchial reactivity to inhaled methacholine (3h post-exposure). Inflammation and obesity markers were assessed in the blood (pre, 4h post, and 20h post exposures) and induced-sputum (4h post-exposures and on 24h pre-exposure training day, no exercise): measures of C reactive protein (CRP) (blood only), leptin (blood only), adiponectin, interleukins IL-6, IL-1b, and IL-8, and tumor necrosis factor alpha, and sputum cell differential cell counts. The pre- to post-exposure decrease in forced vital capacity after ozone (adjusted for the change after air exposure) was significantly greater in the obese group (12.5+/-7.5 vs. 8.0+/-5.8%, p<0.05). Post ozone exposure, 6 obese and 6 non-obese subjects responded to methacholine at ≤ 10mg/ml (the maximum dose); the degree of hyperresponsiveness was similar for the two groups. Both BMI groups showed similar and significant ozone-induced increases in sputum neutrophils. Plasma IL-6 was increased by exercise (4 hr post air exposure vs. pre) only in the obese but returned to pre-air exposure levels at 20hr post-exposure. Plasma IL-6 was significantly increased at 4hr post ozone exposure in both groups and returned to pre-exposure levels by 20h post-exposure. These results confirm our previous findings of greater post-ozone spirometric decrements in obese young women. However, acute ozone-induced airway reactivity to methacholine and airway inflammation did not differ by obesity at the exposure and exercise levels used.

## Introduction

Obesity is recognized as a state of chronic low-grade systemic inflammation. Adipose tissue secretes a number of inflammatory adipokines (e.g. cytokines such as TNF-a and IL-6, and hormones leptin and adiponectin) into the blood that may modulate inflammation throughout the body including the lungs [1]. Obesity also appears to increase non-specific airway responsiveness [1–4]. Mouse models suggest that obesity enhances airway responses to ozone air pollution. Shore et al [4] showed that leptin-deficient obese mice had greater airway hyperreactivity to intravenous methacholine following ozone exposure compared to lean, wild-type mice. Furthermore, administration of exogenous leptin (which is increased in the serum of obese individuals) was shown to enhance ozone-induced cytokine and protein release into bronchoalveolar lavage (BAL) fluid of lean, wild type mice [4].

In a human chamber exposure study we showed that ozone-induced lung function decrements (forced expiratory volume in one second, FEV1) were greater in obese vs. non-obese women [5]. While our study population was predominantly normal weight, we found that higher body mass index may be a modest risk factor for adverse lung function effects associated with ozone exposure, especially for women. Reduced lung volumes and altered breathing patterns in the obese may also contribute to their enhanced airway reactivity to inhaled irritants [6]. Stretching of airway smooth muscle during tidal breathing and especially during deep breaths, i.e. sighs, acts as a potent bronchodilator that might ameliorate ozone-induced bronchoconstriction. The increased chest load associated with obesity may also diminish tidal breathing volumes and frequency of sighs during ozone exposure.

Does obesity affect ozone-induced changes in airway function, reactivity, and inflammation in young, adult women? The purpose of the current study was to compare the effect of controlled, ozone chamber exposure in obese vs. non-obese female adults. Based on our previous study [5], where we found evidence for a greater effect of BMI on ozone-provoked lung function changes in women vs. men, we chose to study two distinct BMI (body mass index) groups of young non-smoking females (BMI ≤ 25 kg/m2, Normal weight, and 30 kg/m2 ≤ BMI ≤ 40 kg/m2, Obese). While BMI reflects general obesity, waist circumference reflects abdominal obesity that is more strongly related to several adverse health outcomes than BMI [7] and may therefore be relevant to the possible effect of obesity on ozone response. Consequently, we also used waist circumference as a secondary criterion for our two study groups. Finally, as it has also been shown that the responsiveness to ozone decreases with age in adults [5, 8] we also limited our study subjects to age 18–35.

## Study Design and Methods

The study protocol was approved by the School of Medicine Committee on the Protection of the Rights of Human Subjects of the University of North Carolina. Informed written consent was obtained from all subjects before their participation in the study.

### Study Subjects

Nonsmoking women between the ages of 18–35 years of age were recruited for the study based on body mass index (BMI) and waist circumference; twenty obese (BMI 30–40 kg/m^2^, waist circumference ≥ 35in) and 20 normal weight (BMI < 25 kg/m^2^, waist circumference ≤ 29.5in). Percent body fat was measured by bioelectric impedance analysis (BIA) (Quick Medical, Snoqualmie, Wash.). We attempted to match ethnic composition between the obese and normal weight cohorts as closely as possible. Subjects participating in the study underwent a thorough medical screening evaluation, including physical examination. Subjects were excluded if they had any chronic medical condition considered as a contraindication to the exposure study, e.g., significant cardiovascular disease, diabetes requiring medication, chronic renal disease, or chronic thyroid disease.

Baseline lung function and volumes were measured by spirometry and body plethysmography. Subjects were required to have a ratio of FEV1 to forced vital capacity (FEV1/FVC) ≥ 70%, as well as an FEV1 > 75% of predicted normal for height and age. They were excluded if they had any history of asthma, chronic respiratory disease, or had active allergic rhinitis. A positive methacholine response on the training day, defined as a 20% or greater reduction in FEV1 after inhalation of a concentration less than 2.5 mg/ml methacholine (procedure described further below) excluded subjects as having mild to severe airway hyper-responsiveness. During the course of participation in the training and exposure protocols all subjects were required to abstain from; ingestion of multivitamins; other vitamins including Vitamins C or E or carotenoids; aspirin or any other anti-inflammatory medications (including nasal steroids); anti-histamines, exposure to cigarette smoke or other irritants. Because there has been some data suggesting that cigarette smoking reduces responsiveness to ozone [9], subjects who had smoked any type of tobacco within the past 30 days and those who had smoked greater than 5 pack-years lifetime were excluded from the study. We also performed a cotinine assay (Bio- Quant, Inc., San Diego, CA) on subject’s pre-ozone exposure urine sample to determine if they had recent cigarette smoke exposure inconsistent with their reported smoking history.

### Study Design

The obese and normal weight subjects were exposed to both filtered clean air and ozone (0.4 ppm, 2hr) in the USEPA human exposure chambers [10] using a double-blinded random cross-over design with exposures separated by a minimum of two weeks and maximum of 6 months. Table 1 provides a timeline of procedures associated with the two exposures. During exposures, subjects performed four 15-minute periods of light exercise (target minute ventilation Ve = 25 L/min, range 20–30 L/min) on a treadmill, each separated by 15 minutes of seated rest. Minute ventilation was monitored during the exercise periods and the treadmill adjusted accordingly to assure maintenance of the target ventilation. Continuous measures of breathing pattern (tidal volume and respiratory rate) were made by respiratory inductance plethysmography. Before and immediately after exposures, lung function (spirometry and body plethysmography) was measured. Symptoms were assessed immediately after each exposure. The subjects were asked to rate 15 different symptoms on a five-point scale ranging from 0 (none) to 4 (most severe). Total symptom severity was obtained by adding scores of all symptoms. Methacholine challenge and sputum induction were performed 3 and 4 hours post exposure respectively. Venous blood, collected for the harvest of plasma (EDTA treated), was drawn pre exposure, and post 4 hours and 20 hours, and induced-sputum was collected 4 hours post-exposure. The plasma and sputum supernatants were stored at -80°C and were subsequently assayed for concentrations of C- reactive protein (CRP), leptin, adiponectin, IL-6, IL-1β, IL-8, TNF-a, and sputum cells were analyzed for differential cell counts.

**Table 1:** Study Design Timeline (Exposure Day).

Study Steps	Procedures	Time required
**Pre-Exposure**	Medical evaluation by nurse Urine sample Venous Blood sample Lifeshirt Vest on (calibration) Spirometry Body Plethysmography	1hour
**Ozone/Air Exposure in the chamber**	Alternating 15 minute periods of exercise on treadmill (Ve = 20–30 L/min) and rest for 2 hour exposure.	2hours
**Post Ozone/Air Exposure- immediately**	Medical evaluation/Symptoms SpirometryBody Plethysmography Lifeshirt Vest calibration	40minutes
**2–4 hours post exposure**	Methacholine challenge Sputum induction Venous blood sample	2hour
**App. 20 hours post exposure**	Medical evaluation Spirometry Venous blood sample	1hour

On a training (no exercise) day prior to the first exposure day, spirometry, methacholine challenge, and sputum induction were performed to test the subject for inclusion/exclusion and to compare these endpoints to those associated with the combination of exercise and exposure.

### Methacholine challenge

Subjects inhaled increasing concentrations of methacholine (starting with an initial normal saline inhalation without methacholine, followed by eight doubling doses from 0.078 mg/ml to 10 mg/ml of methacholine) from a Devilbiss 646/Rosenthal dosimeter, with five inhalations at each concentration. Following each escalating dose, forced expiratory volumes were determined by spirometry to determine if a reduction in forced expiratory volume in 1 second (FEV1) had occurred. Testing was stopped when the FEV1 fell by at least 20% from the post saline value (i.e. the PD20 methacholine dose) or if the highest concentration (10mg/ml) had been inhaled. Subjects who had a PD20 of less than 2.5 mg/ml on the training day were considered to have significant airway hyperresponsiveness by ATS guidelines [11] and excluded from participating further in the study. Subjects were not excluded from further participation if the PD20 fell below 2.5 mg/ml on the exposure days since increased methacholine responsiveness was a primary endpoint of interest in this study. However, any subject with a baseline FEV1 of less than 70% predicted immediately prior to the start of challenge did not continue with the challenge. Regardless of whether a subject experienced bronchoconstriction associated with the methacholine challenge, we had each subject inhale 2 puffs of albuterol from an MDI at the conclusion of the methacholine challenge in order to maintain consistency among all subjects with regard to possible effects of albuterol on subsequent sputum induction endpoints.

### Continuous breathing patterns

On each exposure day the subject was fitted with a Lifeshirt vest (Vivometrics, Inc., Ventura, CA) that allowed continuous measurements of breathing pattern (tidal volume and respiratory rate) by respiratory inductance plethysmography [12]. The changes in inductance associated with chest and abdominal expansion/contraction were calibrated to a fixed 800ml volume prior to exposure and compared/corrected to the mean pneumotachograph volume measurements during the exercise periods. Average tidal volume, breathing rate, minute ventilation, and frequency of sighs were analyzed from the Lifeshirt recorder post-exposure. A sigh was defined as any breath that was more than 2.5 times the median tidal volume. The study coordinator monitored cough frequency during the exposures.

### Induced sputum and cell differentials

Sputum induction and processing were performed according to previously published methods [13]. In brief, subjects inhaled increasing concentrations (3%, 4%, and 5%) of hypertonic saline for 7 minutes each, for a total of 21 minutes. Manually selected sputum plugs were weighed and treated with 0.1% dithiothreitol (DTT) for cell and mucus dispersion. Following centrifugation, differential cell counts were analyzed from (Diff-Quik)–stained slides, based on 400 cells, and expressed as a percentage of total non-squamous epithelial cells. Sputum samples contained a minimum of 120,000 total cells for analysis a differential cell count containing less than 40% squamous epithelial cells, and cell viability of at least 50%, thus minimizing variability in cell recovery and squamous epithelial cell contamination.

### Blood and Sputum Biomarkers

Pre and post-exposure plasma and induced-sputum supernatants were analyzed by immunoassay for concentrations of CRP, leptin, adiponectin, IL-6, IL-1β, IL-8, TNF-a on an Imager 2400 (Meso Scale Discovery, Gaithersburg, MD), using human-specific reagents obtained from the instrument manufacturer. The validated kits are designed and developed to provide both high performance and consistency within and between kit lots (V-PLEX (Mesoscale Discovery) utilizing standard curves for all assays [14]. All sample analyses were performed in duplicate and averaged to provide the final measure associated with a given subject and exposure condition. The plasma samples were evaluated using the methods provided with the reagents. Sputum supernatant analyses were performed following an additional sample predilution step as recommended by the reagent manufacturer, i.e. PBS plus 1% BSA (IL-6, IL-1β, IL-8, TNF-a) or the provided 1% blocker A solution (CRP) or the provided diluent 100 (adiponectin).

### Statistical Analysis

Group comparisons were made by independent samples t-test and differences between clean air and ozone challenge by paired analysis (P < 0.05 for two tailed tests). We calculated sample size based on our a priori primary endpoint, methacholine reactivity, the most significant endpoint associated with the obese mouse model studies [4]. In estimating the sample size required to be confident that ozone yields a significantly greater reactivity to methacholine for obese vs. non-obese, we used published data on methacholine response in non-obese subjects following an ozone exposure similar to that proposed here (0.4ppm for 2 hours) [15]. In that study the mean log reduction in methacholine dose (a dose required to double airway resistance) for post vs. pre ozone exposure was 0.52 +/-0.49. Based on the mouse model studies, we hypothesized that we would observe a log reduction of 1.0 in the obese population. Assuming a similar SD in the obese as was observed in the non-obese data we estimated that n = 20 in each group would achieve more than adequate power (90% for one tailed test, p<0.05) (n = 15 required for 80% power). The exploratory nature of the study and the limited human data in obese subjects did not allow for accurate power calculations on the full range of response variables that we assessed, but the sample sizes should have afforded us sufficient data to ascertain meaningful differences between obese and non-obese for these exploratory endpoints.

## Results

Table 2 summarizes the subject characteristics and baseline lung function values for the obese vs. normal weight females. On their ozone exposure study two subjects (one obese, one normal weight) were found to have high pre-exposure urine cotinine levels (both >370 ng/ml) consistent with heavy cigarette smoke exposure [16] and were therefore eliminated from the summary data analysis. All other subjects had urine cotinine concentrations < 60ng/ml which is consistent with them being nonsmokers and the levels did not differ significantly between obese and normal weight groups (N = 19 in both).

**Table 2:** Summary mean (SD) of subject anthropometric and baseline lung function data.

	Obese (n = 19)	Normal weight (n = 19)	P value
Age	27.7 (5.2)	24.4 (3.7)	< 0.05
Race	9AA, 8C, 2H*	10AA, 8C, 1A*	
Ht (cm)	165 (5)	166 (8)	
Wt (kg)	95 (10)	60 (8)	< 0.0001
BMI (kg/m^2^)	34.6 (2.6)	21.7 (1.4)	< 0.0001
Waist size (cm)	97.5 (4.8)	69.6 (3.8)	< 0.0001
Body fat (%)	43.9 (4.1)	24.7 (5.2)	< 0.0001
FVC (liters)	4.10 (0.44)	3.82 (0.56)	0.09
FEV1 (liters)	3.38 (0.41)	3.20 (0.58)	
TLC (liters)	5.07 (0.53)	4.76 (0.68)	
FRC/TLC	0.40 (0.11)	0.52 (0.06)	< 0.0005
sGaw (l/cm-H_2_O-sec)	0.172 (0.068)	0.192 (0.067)	

*AA = African American, H = Hispanic, C = Caucasian, A = Asian

Both groups showed significant decrements (pre—post exposure) in FVC, FEV1, IC (inspiratory capacity), and sGaw (specific airway conductance) for ozone vs. clean air exposure (p < 0.05). No other ozone-induced changes in spirometric or plethysmographic variables were observed. The mean decrements in lung function variables (the difference in % fall (pre-post) between ozone and air exposures) for obese and normal weight females are summarized in Table 3. Only the decrements in FVC were statistically different between obese and normal weight females (Table 3), i.e. the obese had a greater decline in FVC following ozone vs. clean air exposure. Interestingly, while the ethnic composition was similar in the two groups the African-American (AA) obese tended to have a greater reduction in FVC than obese subjects of other ethnicities (Caucasian, Hispanic, and Asian) (delta % fall in FVC = 15.7+/-5.5 and 9.6+/-8.1 for AA vs. others respectively, p = 0.08). Also of note, the slight but insignificant greater TLC in the obese, Table 2, resulted in similar ozone induced % fall in TLC for both groups, 8.8% (obese) and 8.7% (normal), and, consequently, a similar mean absolute post ozone TLC, 4.50 (obese) and 4.49 (normal) liters.

**Table 3:** Mean (SD) decrements in lung function variables (delta % fall) expressed as ((Pre-Post Ozone)/Pre Ozone)–(Pre-Post Air)/Pre Air)) X 100.

	FVC	FEV1	IC	sGaw
Obese	12.5 (7.5)	15.9 (8.6)	16.7 (14.2)	12.6 (26.2)
Normal Wt	8.0 (5.8)	11.7 (7.1)	10.2 (13.2)	12.4 (21.0)
P	< 0.05	0.11	0.12	NS

Mean total symptom score post-ozone was low for both groups and not different (4.7 +/-5.4 and 3.5 +/-2.9 for normal weight and obese respectively). The obese subjects tended to report more pain on deep inspiration (PDI) post ozone exposure (11/19 (obese) vs. 5/19 (normal) reported nonzero responses: mean response = 0.9 +/- 0.9 (obese) vs. 0.4 +/- 0.8 (normal), p = 0.10). Within the obese group the mean delta % fall in FVC tended to be greater in those reporting PDI post-ozone exposure (14.5 +/- 8.2 vs. 9.3 +/- 6.4) though the difference was not statistically significant (p = 0.16). Within the normal group there was no difference in delta % fall in FVC for those reporting PDI vs. those that did not. Finally, the normal weight group tended to report greater cough severity post-ozone exposure compared with the obese group (1.05 +1.22 vs. 0.47 +/- 0.84 respectively, p = 0.10).

Only one obese subject was reactive to methacholine on the training day, i.e., PD20 ≤ 10 mg/ml methacholine inhalation. Post-exposure (3 hours) methacholine reactivity results for clean air and ozone exposure and each study group are shown in Table 4. There was no difference in the number of responders (those subjects who demonstrated airway reactivity to methacholine in response to exposure) or in the PD20 for responders between the obese and normal weight subjects for either the ozone or clean air exposures. The obese subject who responded at 0.625 mg/ml after the clean air exposure was not challenged on the ozone exposure day for safety reasons. This subject’s FEV1 had decreased 30% immediately post-ozone exposure and had not returned to a greater than 70% predicted by 3 hours post-exposure (the assigned time for the methacholine challenge). For both study groups, those individuals who were responders post-clean air exposure were also responders after ozone exposure.

**Table 4:** Methacholine reactivity. Responder defined by 20% fall in FEV1 at ≤ 10mg/ml. PD20 = dose at which FEV1 falls by ≥ 20%.

	Number of responders	PD20 (mg/ml)
Obese		
Post clean air	2	0.625 and 5
Post ozone *	6	5.5 +/-3.8
Normal weight		
Post clean air	2	10 for both
Post ozone	6	6.5 +/-4.0

* N = 18, one subject excluded for safety reasons (see text).

The resting and exercise breathing parameters associated with the exposures are given in Table 5. The mean treadmill speed to achieve similar exercise minute ventilation in the normal vs. obese group was significantly different (3.3+/-0.3 vs. 2.4+/-0.4 mph respectively, p < 0.0001). There were no significant differences in breathing parameters between obese and normal weight subjects except for the number of sighs during exercise on the ozone exposure day, with significantly more sighs (two-fold) in the normal weight females. These sighs (at 2.5-fold the median tidal volume) during exercise are essentially inspiratory capacity (IC) breaths for each group. The number of coughs during the exposures was low and there were no differences between the two study groups during the ozone exposure, 2.6+/-5.4 (obese) and 4.9+/-6.5 (normal weight). Only the normal weight group had increased cough frequency for ozone vs. clean air exposure (4.9+/-6.5 vs. 0.5+/-0.9 respectively, p < 0.01). These cough data were consistent with the post-ozone symptom reporting for cough severity.

**Table 5:** Breathing patterns (mean (SD)) measured by respiratory inductance plethysmography. N = 18 for obese, one subject’s data was not recorded.

	Ve (L/min)		Tidal volume (ml)		Breathing freq (per min)		Number of sighs	
	Rest	Exercise	Rest	Exercise	Rest	Exercise	Rest	Exercise
**Obese**								
Ozone	8.4 (1.9)	25.8 (2.7)	424 (96)	946 (217)	20 (3)	31 (7)	19 (8)	12 (8)
Clean air	7.9 (1.7)	25.0 (2.9)	414 (114)	940 (287)	20 (2)	31 (9)	17 (10)	16 (12)
**Normal Wt**								
Ozone	9.2 (2.7)	24.9 (2.3)	468 (126)	917 (168)	20 (2)	29 (4)	22 (14)	25 (21)*
Clean air	9.0 (2.7)	24.9 (2.7)	470 (131)	950 (152)	20 (2)	28 (4)	19 (10)	20 (17)

* P < 0.05 for obese vs. normal weight

Twelve (12) obese and 12 normal weight subjects produced adequate sputum samples (see methods) for analysis of cell differentials on the training and both exposure days. Both groups showed a significant ozone-induced increase in sputum neutrophil concentration and as % of total recovered cells, Table 6. No differences in neutrophil concentrations were observed between the training and air exposure days, i.e., a comparison of no-exercise to exercise baselines. There were also no differences in sputum neutrophils between groups for either the train visit (no exercise) or the two exposures. Among the measured cytokines in sputum there were no differences by weight group and only IL-6 showed any evidence of ozone-induced increases in either group. There was no exercise effect on sputum IL-6 in either group (i.e. train vs. air study days) but there was a significant increase for ozone vs. air exposure in the normal weight group (Table 6). There was also a trend for an increase in the obese for ozone vs. air (Table 6, p = 0.11 by Wilcoxon matched pairs signed-rank test). There were no group differences on any exposure/train day, possibly due to the large variability for each group. However, among all subjects post-ozone log (sputum IL-6) and log (sputum neutrophil concentration) were strongly correlated (R = 0.75, p < 0.0001).

**Table 6:** Sputum PMNs (concentration and %of recovered cells) and Sputum IL-6 concentration (pg/ml).

	Concentration cells/mg median (25th, 75th %ile)	% of total cells mean (SD)	IL-6 (pg/ml) median (25th, 75th %ile)
Obese Post Ozone	194.5 (25, 390.5)*	48 (28)*	13.1 (3.0, 24.4)
Obese Post Air	62.5 (7.5, 174)	22 (21)	7.9 (1.4, 10.8)
Obese Train	50 (16, 103.5)	19 (16)	2.2 (0.9, 10.1)
Normal Post Ozone	186.5 (15, 257.5)*	39 (25)^	15.9 (6.7, 33.3)^
Normal Post Air	64.5 (27, 100.5)	14 (10)	4.8 (2.6, 9.1)
Normal Train	52 (14, 68.5)	20 (26)	5.7 (1.5, 20.5)

* P < 0.05 vs Post Air and Train by Wilcoxon matched pairs signed-rank test

^ P < 0.05 vs Post Air by Wilcoxon matched pairs signed-rank test.

**Fig 1:**
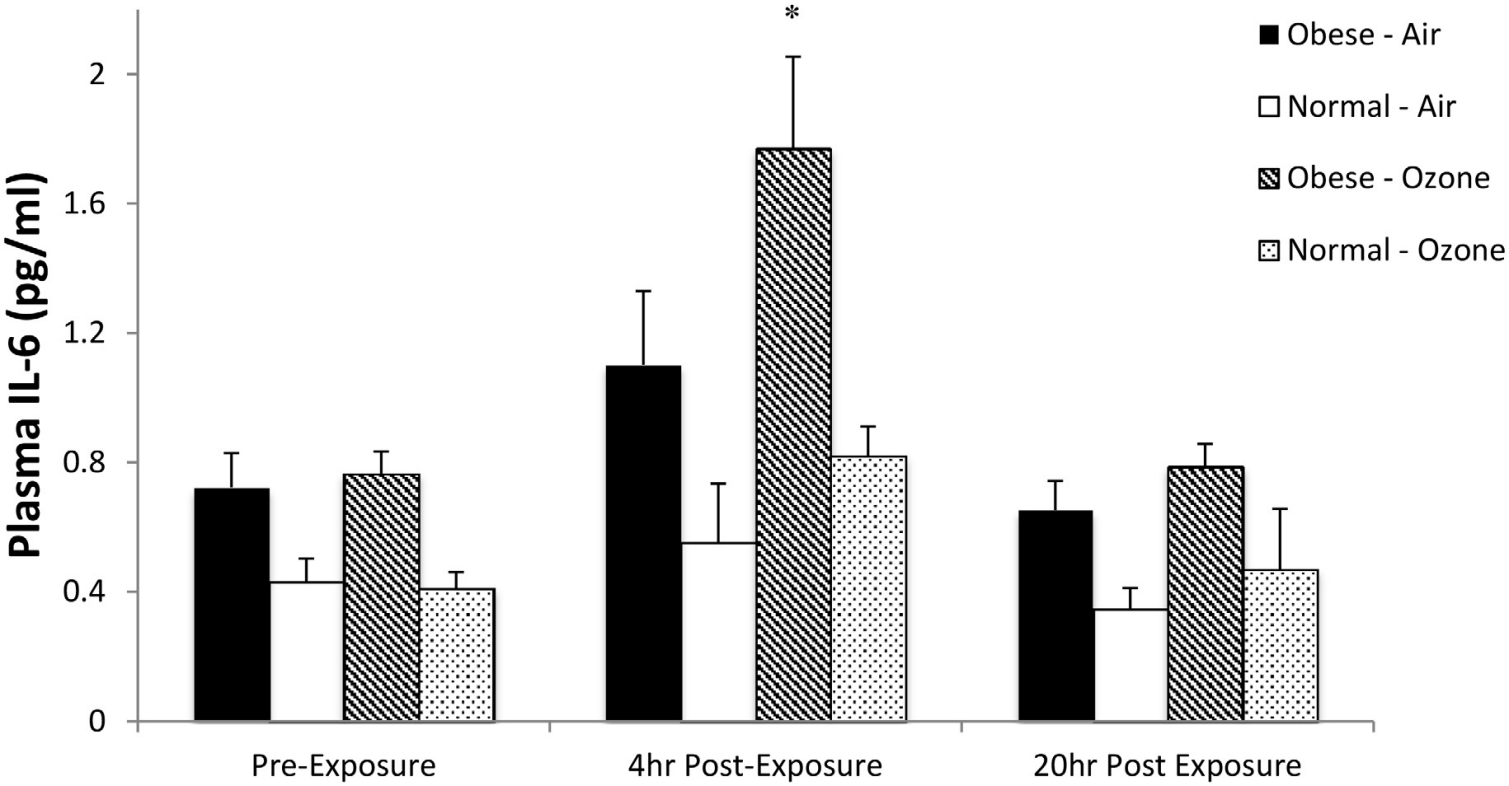
Mean (SE) plasma IL-6 concentrations for pre- vs. post-air and pre- vs. post-ozone exposure. Pre-exposure levels of IL-6 were less in normal weight than obese (Table 7). Both groups show significant increase in IL-6 4-hours post ozone exposure (P < 0.005). *P < 0.006 for Obese vs. Normal 4-hours post-ozone exposure.

Average pre-exposure plasma concentrations of cytokines and adipose hormones for obese and normal weight are given in Table 7. Baseline plasma leptin and CRP were respectively about 5 and 2 fold higher in the obese vs. the normal weight subjects (p < 0.0001 and < 0.05). Levels of the fat hormone adiponectin were less in the obese vs. normal weight (p < 0.05) and the cytokines TNF-α and IL-6 were elevated in the obese vs. normal weight (p < 0.01 and p < 0.0005). Plasma IL-6 showed the most significant changes associated with ozone exposure in either group, Fig 1. There was a trend for plasma IL-6 to be acutely increased by exercise (pre- vs. 4 hr post air exposure) only in the obese (p = 0.06) but returned to pre-air exposure levels at 20h post-exposure. In both groups plasma IL-6 was significantly increased by ozone exposure at 4hr post-exposure (p < 0.005) and returned to pre-exposure levels by 20h post-exposure. In the obese group only there was a comparable decrease for pre- vs. post- air and ozone exposures for plasma levels of leptin and TNF-a (p < 0.05), suggesting an exercise or time-of-day effect on levels of these markers in the obese.

**Table 7:** Summary (mean and SD) of baseline plasma adipose hormones and chemokines (average of two pre-exposure for each subject).

	Leptin (ng/ml)	Adiponectin(ug/ml)	CRP (ug/ml)	TNF-a (pg/ml)	IL-1b (pg/ml)	IL-6 (pg/ml)	IL-8 (pg/ml)
Obese	81.7(34.0)	19.2(6.5)	6.7(5.2)	1.48(0.48)	0.12(0.19)	0.76(0.35)	2.08(2.38)
Normal	16.5(7.6)	23.5(6.3)	3.6(4.1)	1.12(0.25)	0.11(0.08)	0.42(0.15)	1.27(0.65)
P	< 0.0001	< 0.05	< 0.05	< 0.01		< 0.0005	

The complete data set for parameters reported in this manuscript can be found in S1 File.

## Discussion

While a number of mouse model studies have investigated the role of obesity in airway hyperreactivity and responsiveness to ozone [4, 17–19], this is the first controlled human exposure study designed to specifically assess this relationship. Our previous retrospective study [5] was not designed to investigate the effect of obesity on ozone responsiveness, i.e. most of the study subjects were in the normal weight range. Our finding in the current study that obese females had a greater reduction in FVC associated with 0.4ppm ozone exposure (Table 3) is consistent with our previous findings [5]. While the exposure duration was longer in the current study (2 vs. 1 ½ hours) the intermittent exercise was less frequent and at a lower level than our previous study (1/2 vs. 2/3 of time spent in exercise and mean exercise Ve = 25 vs. 32 L/min respectively) and thus the effective exposure was lower in the current study. We set the exercise level in the current study to maintain similar minute ventilation between the two groups and, by extension, ozone dose to the lungs. Consequently, the mean treadmill speed to achieve similar exercise minute ventilation was higher in the normal vs. obese group. Clearly if the obese were to exercise at the same work load as the normal weight individuals they would receive a higher dose of ozone and a greater associated effect to the lungs. It could be argued, however, that in everyday life the obese generally exercise at less effort than non-obese individuals.

It is well established that the decreased FVC and FEV1 associated with ozone exposure is due to an inhibition of maximal inspiration [20]. The primary mechanism associated with the spirometric response to ozone exposure is a reflex nociceptive inhibition of inspiration [21] that likely originates from bronchial C fibers. The trend we observed for the African American (AA) obese females to have a greater ozone-induced reduction in FVC than the obese subjects of other ethnicities (mostly Caucasian) is interesting and deserves further investigation. Seal et al [22] found no difference in ozone responsiveness by spirometry in African Americans compared with Caucasian adults but did not study obesity as a secondary influence. Finally, it was interesting that the post-ozone TLC was so similar between the obese and normal groups, which might suggest an absolute upper limit of inspiration after ozone, though we can think of no clear explanation for this observation.

We were unable to show an effect of obesity on airway reactivity to methacholine for either post clean air or ozone exposures. Ozone exposure resulted in a third of the subjects (Table 4) having a PD20 ≤10mg/ml but no difference between obese and normal weight individuals in number or PD20. Because we excluded subjects who had a history of asthma or had a PD20 of less than 2.5 mg/ml on the training day, i.e. those who had moderate to severe airway hyperreactivity were excluded from participation by design. As a result the subjects enrolled were essentially nonreactive to methacholine at screening. This exclusion, done because of safety concerns at the time of study initiation, may have compromised our power to detect differences in airway reactivity in response to ozone in obese versus normal weight individuals. The findings from a number of studies are mixed on whether obesity is a risk factor for airway hyperreactivity (AHR) in general [23]. A number of chamber studies have shown that ozone exposure enhances AHR [15, 24, 25] in non-asthmatic subjects but mostly by assessing changes in airway resistance (sRaw) with methacholine challenge. It may be that this index of acute airway obstruction is more sensitive to airway hyperreactivity than the spirometric endpoints used in our study. Nevertheless even when we decrease the threshold fall in FEV1 to 10% (i.e. PD10 rather than PD20) the number of methacholine responders only increased to 8 in the obese and to 10 in the normal weight individuals, i.e., no hint at a difference between the two groups. Hiltermann et al also suggested that the time after exposure is critical for detecting ozone-induced AHR [26]. They found increased AHR in both non-asthmatic and asthmatic subjects at 12 hours post-ozone exposure (as compared to the 2–3 hours in our study) and suggested that the effect coincided more closely in time with the peak in ozone-induced inflammation (i.e. influx of neutrophils into the lung). To our knowledge we are the first to assess the role that obesity may play in ozone-induced AHR in non-asthmatics. But a series of studies in a variety of mouse models of obesity have all shown increased airway reactivity to intravenous methacholine in the obese vs. wild type mice following ozone exposure [4, 17–19]. There are several possible reasons for the different findings in mouse vs. human studies, e.g. dosing intravenously vs. aerosol and greater, innate AHR in mouse vs. human. A targeted study of obese vs. normal weight asthmatics may show an effect of obesity on ozone-induced AHR [25].

To our knowledge we are the first to measure continuous breathing of our subjects throughout the chamber exposures using respiratory inductance plethysmography. There were no differences in total ventilation or breathing patterns between the two groups for either clean air or ozone exposures. The only difference we found was in the number of sighs during the exercise periods of the ozone exposure, i.e. fewer for the obese subjects. This finding may reflect the lesser ability for the obese to take a deep a breath (close to an inspiratory capacity breath during exercise) associated with both their greater ozone spirometric responsiveness and the increased mechanical load on their chest wall. It is intriguing to speculate that the number of sighs during the exercise periods of the ozone exposure may have mitigated the inability of subjects to take a deep inspiration during post exposure spirometry, and that this advantage may have been reduced in the obese. There was, however, no correlation between lung function decrements and sigh frequency in either group for the ozone exposure day. Within the obese group there was also no difference in number of sighs for those reporting PDI symptoms vs. those that did not. Nevertheless, these findings on sigh frequency may deserve further study, e.g. determine if post ozone exposure decrements in spirometry may be reduced by incorporating controlled sighs (deep breaths) during the exposure. Ozone-induced cough frequency and severity was also more prevalent in the normal weight individuals suggesting that the larger number of sigh breaths may have provoked coughing in this cohort. But there was also no correlation between sigh and cough frequency in either cohort alone or combined.

Others and we [13, 27] have previously found ozone-induced airway inflammation, as indicated by increased sputum or lavaged neutrophils. While ozone exposure increased sputum neutrophilia in both groups (Fig 1), we were unable to show any differences between the obese and normal weight females post ozone. Neither were baseline (no exercise) or clean air (post-exercise) neutrophils different between the two groups. We did not find ethnic differences in ozone-induced airway inflammation (e.g. airway neutrophils) in our obese subjects compared with normal weight subjects. Sputum cytokines did not differ between the two groups for any of the study days. There was a significant increase in IL-6 for ozone vs. air exposure in the normal weight group and a trend for an increase in the obese that was not statistically significant likely due to the large variability in IL-6 post ozone. The cytokine IL-6, produced by macrophages and epithelial cells in response to ozone [28], has been shown to help regulate the influx of neutrophils into the lung associated with ozone exposure [29]. Thus, it was not surprising that we found a correlation between sputum IL-6 and neutrophils in response to ozone exposure. In the mouse models of obesity lavaged neutrophils and IL-6 were also increased with ozone exposure for both wild-type and obese mice, with a significantly greater increase in IL-6 for the obese mice [4, 18, 19]. Our data (Fig 1) suggest that with larger numbers of sputum producers we might also have seen a greater increase for IL-6 in the obese subjects.

Our finding of increased leptin and decreased adiponectin (pro- and anti-inflammatory adipokines respectively) in the plasma of our obese vs. normal weight individuals was expected [1, 6]. The finding of higher levels of plasma CRP, TNF-a and IL-6 in the obese is also consistent with the findings of others [1,6] suggesting the presence of chronic, systemic inflammation associated with obesity. The only plasma hormone/cytokine that increased with either clean air/exercise or ozone/exercise was IL-6. The increase in IL-6 with exercise in the obese suggested a mild inflammatory response to a moderate level of exercise in these individuals. Ozone exposure increased IL-6 in both groups, further enhancing the levels of IL-6 in the obese compared to normal weight subjects, i.e. the combined effects of exercise and ozone exposure appeared to accentuate the difference in IL-6 levels between the obese and normal weight subjects (Fig 1). Whether such acute increases in systemic inflammation translate into enhanced chronic health effects in the obese in high ozone environments should be investigated further, especially in those with asthma [1].

## Conclusions

The results of this first targeted study addressing the effect of acute ozone-induced changes in airway function, reactivity, and inflammation in obese young adult women suggests that obesity, per se, has minimal effect on the endpoints we measured. In this randomized controlled exposure study, as in our previous retrospective study [5], we found that the obese had a larger reduction in vital capacity associated with the acute ozone exposure compared to normal weight females. Both groups had an expected ozone-induced increase in airway reactivity and inflammation that has been seen in previous chamber studies, but no obesity related difference was observed. We found no correlations between ozone-induced changes in lung function vs. either sputum or blood markers. This finding is consistent with others finding no relationship between increased inflammation and depressed lung function associated with ozone exposure in healthy normal subjects [10, 30]. Future chamber studies might consider targeting obese and normal weight individuals with asthma, i.e. those with both a greater baseline airway reactivity/inflammation and response to ozone [1, 3]. The combined effect of exercise and ozone exposure appeared to enhance the baseline difference in systemic inflammation between the obese and normal weight individuals. Obesity may deserve further attention as a modifying factor in epidemiology studies of the effects of both short-term and long-term exposure to ozone, especially in asthmatics.

## Supporting Information

S1 File Final DataSet. (XLSX)
